# Delayed cerebral infarction in pneumococcal meningitis due to postinfectious vasculitis: A case report

**DOI:** 10.1097/MD.0000000000042624

**Published:** 2025-05-23

**Authors:** Chao Lai, Lin Sun, Min Wang, Lifei Wei, Fang Jiang, Zhichao Bi

**Affiliations:** a The Second Hospital of Shandong University, Jinan, China; b Cheeloo Hospital of Shandong University, Jinan, China.

**Keywords:** delayed cerebral infarction, pneumococcal meningitis, postinfectious vasculitis, stroke

## Abstract

**Rationale:**

Delayed cerebral infarction (DCI) is a rare but devastating complication of bacterial meningitis that is characterized by an initial good recovery followed by sudden deterioration several days after presentation. The potential mechanisms underlying DCI encompass postinfectious vasculitis, vasospasm, and cerebral thrombosis. Sequential magnetic resonance angiography and characteristic high-resolution magnetic resonance patterns are helpful in discerning the cause of DCI.

**Patient concerns:**

A right-handed 45-year-old man with nasal leakage of cerebrospinal fluid from a car accident was admitted to the local hospital for fever, vomiting, and impaired consciousness.

**Diagnoses:**

Post-infectious vasculitis.

**Interventions:**

Meropenem (1 g 3 times/day), vancomycin (0.5 g 4 times/day), and dexamethasone (10 mg/day, gradually reduced to withdraw) were administered immediately after the patient was diagnosed with *Streptococcus pneumoniae* meningitis. After the patient was diagnosed with DCI, we applied nimodipine (10 mg 3 times/day) to prevent vasospasm, clopidogrel (75 mg/day), and heparin to counteract thrombosis, and prednisone (60 mg/day) was applied to reduce the inflammatory reaction.

**Outcomes:**

On the 52nd day, the patient’s magnetic resonance angiography reexamination indicated a continuous expansion of the cerebral infarction area and a worsening of cerebrovascular occlusion. After the prednisone dose was increased, the consciousness disorder ceased to progress.

**Lessons:**

Early detection and treatment are very important for DCI, the sequential magnetic resonance imaging and high-resolution magnetic resonance imaging are conducive to identify the etiology of DCI and providing guidance for treatment. The delayed tapering of glucocorticoids appears to be beneficial for alleviating the severity of DCI.

## 1. Introduction

Cerebral infarction is a devastating complication of bacterial meningitis with an incidence of up to 25%.^[1]^ Delayed cerebral infarction (DCI) is characterized by an initial good recovery followed by sudden deterioration several days after presentation. The most commonly implicated organism is *Streptococcus pneumoniae.*^[2]^ The mechanism of DCI associated with pneumococcal meningitis remains unclear, some hypotheses including postinfectious vasculitis, vasospasm, and cerebral thrombosis have been reported.^[3,4]^ We report the case of a patient of *S pneumoniae*-caused meningitis and DCI. Monitoring with magnetic resonance imaging (MRI) revealed progressive cerebrovascular stenosis. High-resolution vessel-wall imaging (HR-VWI) showed the whole-segment, concentric enhancement of intracranial arteries, suggesting a diagnosis of vasculitis. The imaging findings from this patient support that postinfectious is one of the potential mechanisms of DCI.

## 2. Case presentation

A right-handed 45-year-old man with nasal leakage of cerebrospinal fluid (CSF) from a car accident was admitted to the local hospital for fever, vomiting, and impaired consciousness. On physical examination, his temperature was 38.3 ℃ and his Glasgow Coma Scale (GCS) score was 10/15 (E2V3M5). Neurological examination revealed neck stiffness with no motor or sensible sign of lateralization. CSF analysis revealed leukocytosis (256 white blood cells/µL), an elevated protein level (3522 mg/L), and very low glucose level (0.01 mmol/L). CSF culture yielded *S pneumoniae*, confirming the diagnosis of pneumococcal meningitis. The patient was immediately started on dexamethasone (10 mg/day, gradually reduced to withdrawal over 2 weeks), meropenem (1 g 3 times/day) and vancomycin (0.5 g 4 times/day).

**Figure 1.**
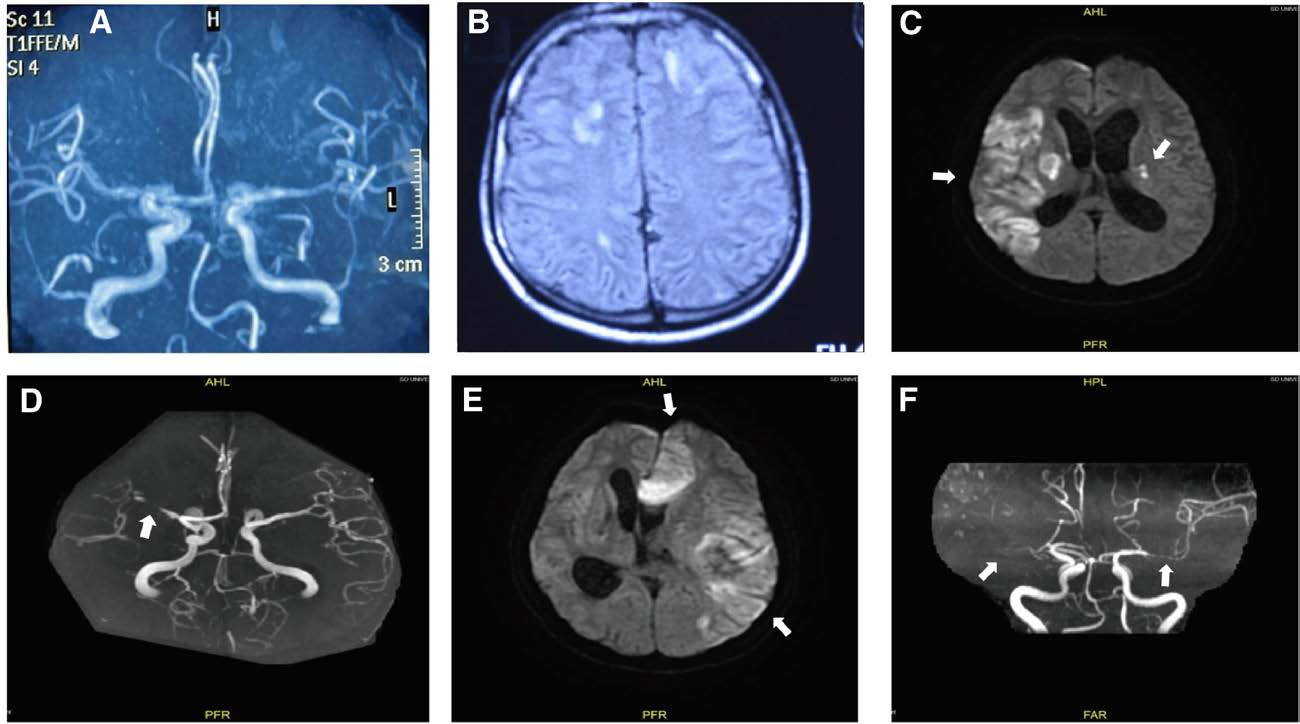
(A) Initial MRA showed there were no significant stenosis of the main cerebral artery. (B) Initial T2 Flair sequence showed scattered cerebral infarctions in both hemispheres. (C, D) The following-up MRI on day 38 showed extensive restricted diffusion in the right hemisphere and patched restricted diffusion in the left hemisphere (white arrows in C), and MRA showed occlusion of the M1 segment of right middle cerebral artery (white arrow in D). (E) Repeat MRI on day 52 showed extensive restricted diffusion in the left anterior and middle cerebral artery territory (white arrows in E). (F) MRA on day 52 showed occlusion of the left ACA and the right MCA, and bead-like stenosis of the left MCA and the right ACA (arrows in F). MRA = magnetic resonance angiography.

On day 5, repeat CSF analysis showed that the patient’s leukocyte count had increased to 1914/µL (83% neutrophils) and that his protein level was 1710 mg/L. The patient improved clinically over the next 2 weeks; his state of consciousness is better than before and he can execute simple commands, with his GCS score improving to 12/15 (E4V3M5) on day 13. On day 14, the patient suddenly began to lapse into unconsciousness and his GCS score dropped to 6/15 (E1V1M3). Subsequently, a brain MRI was performed on the patient on day 15. It was found that there was no significant stenosis in the bilateral middle cerebral arteries, bilateral anterior cerebral arteries, or basilar artery (Fig. 1A). Additionally, the T2 flair sequence showed scattered new infarcts in both hemispheres (Fig. 1B). Repeat CSF examinations performed on days 14 and 34 revealed continuous reduction of the CSF leukocyte count (641/µL and 31/µL, respectively). On days 14, the patient’s CSF protein level was 3701 mg/L; on day 34, this level had decreased to 680 mg/L and blood and CSF cultures were sterile. Repeat lumbar puncture showed that the CSF cell count and biochemical parameters continued to improve, although the patient showed no improvement of consciousness.

**Figure 2.**
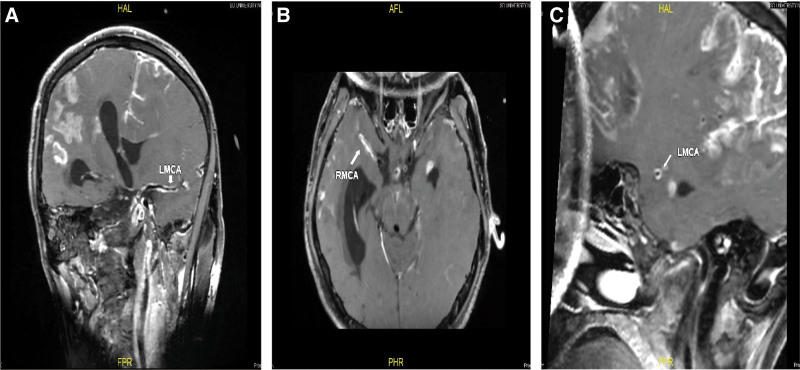
Intracranial vessel-wall images obtained on day 52. (A, B) Coronal images showing orbit-like gadolinium enhancement of the walls of the bilateral middle cerebral arteries (arrows). (C) Sagittal image showing concentric gadolinium enhancement of the wall of the left middle cerebral artery.

The patient was transferred to our hospital on the 37th day of the onset. Repeat neuroimaging on day 38 showed extensive infarcts in the right hemisphere and a patchy infarct in the left hemisphere, with bilateral lateral ventricular dilatation (Fig. 1C). Magnetic resonance angiography (MRA) revealed significant stenosis in the M1 portion of the right middle cerebral artery (Fig. 1D). On day 40, the patient underwent lateral ventricle puncture and drainage to reduce the effect of hydrocephalus on his state of consciousness. Endocarditis was ruled out by echocardiography. Cerebral vasospasm, thrombosis, and vasculitis were considered, and the patient was started on nimodipine (10 mg 3 times/day) and clopidogrel (75 mg/day). Prednisone (60 mg/day) was applied continuously and tapered gradually (reduce the dosage by 1 tablet every 3 days.). On day 52, repeat MRI showed significant enlargement of the infarction (Fig. 1E) and MRA showed occlusion of the left anterior cerebral artery, multiple bead-like stenoses in the M1 segment of left middle cerebral artery, progressive stenosis of the right middle cerebral artery and anterior cerebral artery (Fig. 1F). HR-VWI showed uniform annular centripetal thickening of the bilateral middle cerebral artery walls (Fig. 2A–C). These imaging findings and the course of progressive cerebrovascular occlusion suggested the diagnosis of postinfectious vasculitis. After that, the dose of prednisone was increased to 60 mg and then changed to reducing the dosage by 1 tablet every 2 weeks. Although the patient’s consciousness disorder did not significantly improve during hospitalization, it did not continue to deteriorate either, he was discharged home on the 77th day and remained in a comatose state 3 months after discharge.

## 3. Discussion

Typically, cerebral infarction develops in the first few days of pneumococcal meningitis, when central nervous system inflammation is most severe.^[5]^ The mechanism of DCI is unknown, but thrombosis, a rebound effect of the primary inflammatory reaction initially suppressed by dexamethasone, the activation of coagulation and an immune-mediated para-infectious condition have been suggested.^[3]^ We report the case of a patient with DCI in which continuous neuroimaging monitoring suggested that vasculitis was the underlying cause.

High-resolution MRI uniquely provides the ability to observe changes in vessel walls and luminal structures, and is of great value for etiological diagnosis. In 2017, the American Society of Neuroradiology recommended the use of HR-MRI in clinical practice to distinguish the cause of intracranial arterial narrowing and identify symptomatic, non-stenotic intracranial arterial diseases.^[6]^ Patterns of vessel-wall enhancement and types of vessel involvement have been used to differentiate vasculopathies from intracranial atherosclerotic disease; intracranial atherosclerotic disease generally presents with eccentric thickening and variable enhancement, whereas vasculitis is characterized by smooth, intense, and homogeneous enhancement.^[7]^ In the case reported here, the initial MRA examination revealed no stenosis of the large intracranial arteries. After the deterioration of the patient’s consciousness, HR-MRI revealed infarctions in multiple vascular territories and whole-segment concentric enhancement; sequential MRA images showed progressive vascular occlusion. All of these manifestations led us to diagnose vasculitis.

Diseases mistaken for DCI include reversible cerebral vasoconstriction syndrome (RCVS) and endocarditis. In the present case, we ruled out endocarditis by echocardiography. RCVS is characterized by minimal to no enhancement and minimal wall thickening on postcontrast T1-weighted VWI, and ischemic lesions in RCVS are usually bilateral and manifest in arterial watershed distribution,^[8]^ the pattern of widely spread infraction and the orbital-like significant enhancement of bilateral middle cerebral arteries on postcontrast T1-weighted VWI did not correspond with RCVS. The most importantly, RCVS was diagnosed on the basis of angiographic reversibility or dynamic change, the consecutive neuroimaging at 52-day interval in this patient did not reveal sign of remission, moreover, the patient’s severe neurologic complications which continue to persist, also suggest it is not a self-limiting disease like RCVS.

We reviewed the literature and identified 5 case series and 5 case reports describing 28 cases of DCI complicating bacterial meningitis, but little evidence to support existing hypotheses on the etiology and pathogenesis of DCI.^[2–4,9–14]^ Autopsy studies serve as the gold standard for etiological evidence, and this literature includes 1 such study of 4 patients.^[3]^ Histopathological examination of these patients showed arterial inflammation in all cases and multiple cerebral infarcts,^[3]^ resembling arterial inflammation in acute necrotizing vasculitis with fibrinoid necrosis seen in type III hypersensitivity vasculitis.^[15]^ Distinctive increases in immunoglobulin M and, to a lesser extent, immunoglobulin G deposits have been observed in the arteries of some patients with pneumococcal meningitis, suggesting a role of antigen-induced vasculitis. According to GELL and Coombs classification, type III hypersensitivity takes place when an excess or slight excess of soluble antigens lead to the accumulation of immune complexes, not enough cleared from the circulation by the innate immune system. The MRI presentation of our patient similarly supported the hypothesis of vasculitis. Vasculitis occurs via several inflammatory mechanisms, occurring independently or in combination, including encroachment by inflammatory subarachnoid purulent exudates and infiltration of the arterial wall by inflammatory cells. In the case described here, the patient lapsed into coma on day 14, but his CSF parameters continued to improve for a few days. We postulate that the vascular inflammatory response was not related to bacteria invasion. In previous studies and reports, infarctions involved mainly the penetrating arteries that supply the brainstem and thalamus. In contrast, the present case shows that the large arteries of the anterior circulation can also be involved.

The use of corticosteroids for bacterial meningitis is routine to prevent hearing loss and neurological sequelae, and current European guidelines recommend the initiation of these drugs in all cases of suspected bacterial meningitis.^[16]^ However, corticosteroid administration to patients with DCI may be a double-edged sword.^[2]^ Corticosteroids are known to enhance interleukin-1 mediated calcium dependent vasoconstriction and to reduce levels of vasodilator nitric oxide; which both might contribute to cerebral vasospasms. Schut et al suggested that DCI is associated with the rapid withdrawal of dexamethasone, they suggested DCI may be a rebound effect of the inflammatory response after steroid withdrawal.^[9]^ For this reason, we increased the dosage of glucocorticoids again and maintained it for a certain period after the patient developed DCI. We also administered low-molecular-weight heparin and nimodipine to prevent hypercoagulation and vasospasm. Although all measures that we took failed to halt the occlusion of the intracranial arteries, the patient’s state of consciousness did not continue to worsen after the glucocorticoid dosage was increased again and the withdrawal rate was slowed down. This indicating that the slow tapering of glucocorticoids appears to be effective.

## 4. Conclusion

We postulate that 1 cause of DCI is postinfectious vasculitis, according to the progressive course of vascular stenosis (observed by MRA monitoring) and concentric and long-segmented enhancement patterns seen on postcontrast T1-weighted VWI in the case reported here. This case also suggests that the severity of DCI does not correlate with improvements in CSF parameters. Thus, we suggest that sequential MRI monitoring and postcontrast T1-weighted VWI are effective tools for the early diagnosis of DCI. After the occurrence of DCI, the maintenance application of glucocorticoids appears to prevent the progression of vascular inflammation.

## Author contributions

**Conceptualization:** Chao Lai, Zhichao Bi.

**Formal analysis:** Min Wang.

**Methodology:** Chao Lai.

**Writing – original draft:** Chao Lai.

**Writing – review & editing:** Lin Sun, Min Wang, Lifei Wei, Fang Jiang, Zhichao Bi.
